# Ab initio study of molecular properties of *l*-tyrosine

**DOI:** 10.1007/s00894-023-05648-8

**Published:** 2023-07-13

**Authors:** Roman Boča, Juraj Štofko, Richard Imrich

**Affiliations:** Faculty of Health Sciences, University of SS Cyril and Methodius, 91701 Trnava, Slovakia

**Keywords:** *l*-Tyrosine, Molecular properties, Electronic structure, Ab initio calculations, Molecular descriptors

## Abstract

**Context:**

*l*-Tyrosine is a naturally occurring agent that acts as a precursor in biosynthesis of monoaminergic neurotransmitters in brain such as dopamine, adrenaline, noradrenaline, and hormones like thyroxine and triiodothyronine. While *l*-tyrosine in vacuo adopts the canonical aminoacid form with –NH_2_ and –COOH functional groups, from neutral solutions, is crystallizes in the zwitterionic form possessing –NH_3_^+^ and –COO^−^ groups. As *l*-tyrosine is non-innocent agent with respect to redox processes, redox ability in water expressed by the absolute oxidation and reduction potentials is investigated. The cluster analysis applied to a set of nine related neurotransmitters and trace amines confirms that *l*-tyrosine is mostly similar to aminoacid forms of phenylalanine, octopamine, and noradrenaline.

**Methods:**

The energetic data at the Hartree–Fock MO-LCAO-SCF method has been conducted using def2-TZVP basis set, and improved by the many-body perturbation theory using the MP2 correction to the correlation energy. For the aminoacid form and the zwitterionic form of *l*-tyrosine, a set of molecular descriptors has been evaluated (ionization energy, electron affinity, molecular electronegativity, chemical hardness, electrophilicity index, dipole moment, quadrupole moment, and dipole polarizability). The solvent effect (CPCM) is very expressive to the zwitterionic form and alters the sign of the electron affinity from positive to negative values. In parallel, density-functional theory with B3LYP variant in the same basis set has been employed for full geometry optimization of the neutral and ionized forms of *l*-tyrosine allowing assessing the adiabatic (a) ionization/affinity processes. The complete vibrational analysis enables evaluating thermodynamic functions such as the inner energy, enthalpy, entropy, Gibbs energy, and consequently the absolute oxidation and reduction potentials. Of applied methods, the most reliable are B3LYP(a) results that account to the correlation energy and the electron and nuclear relaxation during the ionization/affinity processes.

**Supplementary Information:**

The online version contains supplementary material available at 10.1007/s00894-023-05648-8.

## Introduction

**Fig. 1:**
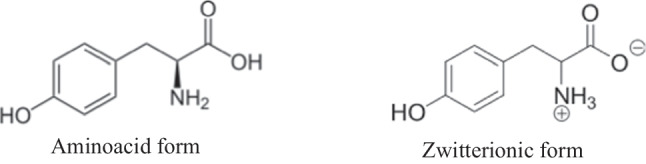
Structural formula of *l*-tyrosine in aminoacid and zwitterionic forms

*l*-Tyrosine is an organic molecule (4-hydroxyphenylalanine or l-2-amino-3-(4-hydroxyphenyl)propanoic acid, C_9_H_11_NO_3_) consisting of the hydroxyphenyl ring and alanine residuum (Fig. 1). It belongs to the non-essential -aminoacids possessing a polar side group. In neutral (physiological) pH, it exists in a zwitterionic form (hereafter Z-form) where the carboxylic oxygen is deprotonated in gain of the ammonium site. Data from Cambridge Crystallographic Database confirms that *l*-tyrosine crystallizes as a zwitterion [1].

**Fig. 2:**
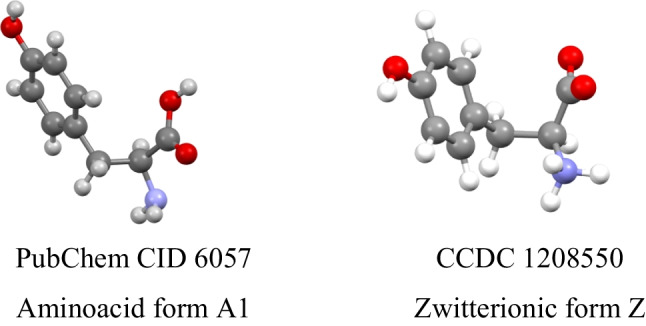
Structural forms of *l*-tyrosine. Color code: gray – C, white – H, blue – N, red – O. Notice differences in the torsion angles C–C-O–H at HO-Ph moiety

According to the neutron diffraction, the molecular structure of *l*-tyrosine in the Z-form is somewhat wrapped [2]; the PubChem database reports a more open aminoacid form (Fig. 2) [3]. The structural versatility is given by 3 rotatable C–C bonds and the number of conformers and/or rotamers is enlarged by the positions of hydrogen atoms in –OH, –COOH, –NH_2_, or –NH_3_^+^ groups.

Some experimental data cover the acidity constants p*K*_a1_ = 2.20 (acid), p*K*_a2_ = 9.11 (base), p*K*_a3_ = 10.07 (phenol group), and the octanol–water partition coefficient log*P* = –2.26. Thus *l*-tyrosine is hydrophobic, however more hydrophilic relative to the structurally analogous phenylalanine (log*P* = –1.38) (Notice, hydrophilic agents with low *P* are found in aqueous sites like blood serum as opposite the hydrophobic substances that prefer the lipid bilayers.) The solubility of *l*-tyrosine in water is *s* = 0.479 g dm^−3^ at standard conditions. Electrochemical studies of *l*-tyrosine were done in aqueous solutions and the voltammetric data are at the disposal [4, 5].*l*-Tyrosine is a proteinogenic amino acid serving also as a precursor for synthesis of thyroid hormones and catecholamines such as dopamine, noradrenaline, and adrenalin. The synthesis of these monoamine neurotransmitters is regulated by tyrosine hydroxylase activity, the rate-limiting step of the reaction converting *l*-tyrosine to *l-*3,4-dihydroxyphenylalanine. L-tyrosine oral ingestion induced transient elevation of the catecholamines in periphery [6]. Recently, effects of dietary of *l*-tyrosine on behavior and cognition showing no significant effects of the amino acid single loading session on exercise performance has been reviewed [7]. In contrast, cognitive studies found that tyrosine loading counteracts decrements in working memory and information processing that are induced by demanding conditions such as exposure to cold temperature or cognitive load [8–10]. Furthermore, long-term hypertyrosinemia induced by nitisinone, a drug which block tyrosine degradation pathway, shows no cognitive decline or increased severity of depression in patients with alkaptonuria [11].

A conformational behavior of tyrosine in different microenvironments in vivo may affect its chemical properties. In particular, phenylalanine and tyrosine were showed to form a cytotoxic amyloid-like assemblies, which may play a role in development of neurodegeneration [12, 13]. Interestingly, corneal keratopathy develops in approximately 10% of alkaptonuria patients, presumably due to tyrosine crystallization during nitisinone-induced hypertyrosinemia [14]. To better understand the role *l*-tyrosine chemical properties a detailed description of electronic structures in different environments is necessary.

The aim of the present study is to get a set of novel molecular descriptors that characterize the electronic structure of the tyrosine molecule in the amino-acid and zwitterionic forms, *in vacuo* and/or water as a solvent. Nowadays, high-quality ab initio calculations that include a part of the correlation energy can be effectively used in studying the molecular properties of bioactive species.

## Methods

For evaluation of molecular properties of *l*-tyrosine the ab initio method was utilized using ORCA package [15–17] and/or Hyperchem software [18]. The basis set of def2-TZVP (valence triple-zeta polarization) quality has been applied consisting of 469 basis functions formed of primitive Gaussians according to the contraction scheme H-{311/1} and C-, N-, O-{62111/411/11/1} where numbers separated by slash refer to the s-, p-, d- and f-shells. The Hartree–Fock (HF) method of molecular orbitals (MO) formed by a linear combination of atomic orbitals (LCAO) in the self-consistent field (SCF) has been improved by applying the many-body perturbation theory in the 2nd-order with the Moller–Plesset partitioning (abbr. MP2) in order to include a part of the correlation energy.

The solvent effect has been considered by exploiting the Conductor-like Polarizable Continuum Model (CPCM) [19]. This was applied with parameters of water (electric permittivity _r_ = 80.4). Consequently, the solvent effect is expressive for energies of molecular ions as well as for the electric dipole moment *p* of neutral molecule (*p* is undefined for ions). The inclusion of the solvent effect does not affect the time consumption calculations visibly.

As a first step, the complete geometry optimization has been performed at the HF-SCF level of calculations. The molecular properties cover the energies of the HOMO (the highest occupied molecular orbital) and LUMO (the lowest unoccupied molecular orbital), the permanent dipole moment *p*, the isotropic quadrupole moment *Q* and the isotropic dipole polarizability (one-third trace of the polarizability tensor _*ij*_ = d*p*_*i*_/d*E*_*j*_ defined as a derivative of the dipole moment component *p*_*i*_ with respect to the intensity of electric field *E*_*j*_). The complete vibrational analysis allows evaluation of the partition function and its electronic, vibrational, rotational, and translational components. Consequently, the standard inner energy *U*^ø^, enthalpy *H*^ø^, total entropic term *T*^ø^*S*^ø^ and the Gibbs energy *G*^ø^ were evaluated. Absolute redox potential is calculated with the help of the reaction Gibbs energy on oxidation and/or reduction: *E*_abs_^ø^(L^0^/L^*q*^) [V] = – _r_*G*^ø^[J mol^−1^]/*zF*, for *F* = 96,485 C⋅mol^−1^ and *z* = 1.

The energetic quantities were handled in more detail. Instead of the HOMO/LUMO data, the vertical ionization energy *E*_i_ and electron affinity *E*_eg_ were calculated in a more sophisticated way, upon the positively and/or negatively charged open-shell system in the UHF (unrestricted Hartree–Fock) variant: *E*_i_ = *E*^+^ – *E*^0^ and *E*_eg_ = *E*^−^ –*E*^0^. They were used in evaluating the chemical potential (approximated by the Mulliken electronegativity $-\mu ={\chi }_{M}=\left({E}_{i}-{E}_{eg}\right)/\left.2\right)$ and the chemical hardness according to Pearson ${\eta }_{\text{P}}=({E}_{\text{i}}+{E}_{\text{eg}})/{2}$. These quantities are regarded as novel molecular descriptors that reflect the electronic gradient and the electronic force constant with respect to change the electron density [20, 21]. The electrophilicity index is introduced as $\omega ={\mu }^{2}/2{\eta }_{\text{P}}$; this electrophilic power is an analogue of the classical electricity *W* = *V*^2^/*R* [22].

Finally, the 3D contour map of the molecular electrostatic potential is plotted on the isovalue surface of charge density [23–26]. The electrostatic potential (against a unit charge) indicates the sites with execs or deficiency of the electron density and thus it indicates sited suitable for electrophilic or nucleophilic interactions along the molecule.

In parallel, also the density-functional theory with the hybrid B3LYP variant has been employed for full geometry optimization of the neutral and ionized forms of *l*-tyrosine.

## Results and discussion

The calculated molecular properties of *l*-tyrosine are listed in Table 1 for the aminoacid form. A comparison of data listed in Table 1 calculated in vacuo and in water brings these findings: (i) HOMO and LUMO energies are not solvent-dependent [items 1, 2]; (ii) the solvent lowers the total energies of the molecule and its ions [items 3–5] and the hydration energy of the neutral molecule is about − 15 kcal mol^−1^; (iii) the solvation reduces the ionization energy and electron affinity that can alter from positive to negative values [items 6, 7]; (iv) the Pearson (chemical) hardness is lowered as well [item 8]; (v) dipole moment rises substantially in the solvent but the isotropic quadrupole moment is solvent-insensitive [items 10, 11]; (vi) the solvation increases the dipole polarizability [item 12]; (vii) the vibrational, rotational and translational contributions to thermodynamic functions at the standard temperature are rather insensitive to solvation [items 15–17, 20–23]; (viii) the solvation influences the electronic contribution to *U*, *H*, and *G* [items 18, 19, 24]. It is seen that the MP2 correction dramatically changes the values of the vertical ionization energy and electron affinity [items 6 and 7].

**Table 1:** Calculated molecular properties of* l*-tyrosine using HF + MP2 and def2-TZVP basis set^a^

		Aminoacid form A1		Aminoacid form A1		Zwitterionic form	
*Molecular properties*		In vacuo		In water		In water	
Item		ΔSCF	+ MP2	ΔSCF	+ MP2	ΔSCF	+ MP2
1	Energy of HOMO	− 196		− 195		− 197	
2	Energy of LUMO	73		74		72	
3	Energy *E*^+^, frozen str	− 392,977	− 394,538	− 393,042	− 394,599	− 393,041	− 394,601
4	Energy *E*^0^, opt str	− 393,143	− 394,744	− 393,159	− 394,759	− 393,160	− 394,763
5	Energy *E*^−^, frozen str	− 393,092	− 394,703	− 393,157	− 394,767	− 393,158	− 394,772
6	Ionization energy *E*_ion_ (v)	166	206	117	160	118.3	162.7
7	Electron affinity *E*_eg_ (v)	51	41	2	− 8	1.3	− 8.3
8	Mol. electronegativity χ_M_ (v)	57	82	57	84	58.5	85.5
9	Chemical hardness ƞ_P_ (v)	108	123	59	76	59.8	77.2
10	Dipole moment *p*/debye	1.181	1.176	2.189	2.149	16.31	15.72
11	Quadrupole moment *Q*/e*a*_0_^2^	− 56.0	− 55.9	− 56.4	− 56.1	− 55.8	-55.6
12	Dipole polarizability α/*a*_0_^3^	110.6	116.4	151.6	160.9	150.9	160.8
13	Solvated surface area *S*/*a*_0_^2^			803		784	
14	Solvated volume *V*/*a*_0_^3^			1449		1441	
15	*E*_vib_(ZPE) – zero point energy	130.0		129.7		131.0	
16	Overall *E*_vib_(*T*^ø^) contribution	135.6		135.3		138.2	
17	*E*_rot_ = *E*_trs_ contribution	0.89		0.89		0.89	
18	Inner energy *U*^ø^	− 39,3006		− 39,3022		− 39,3022	
19	Enthalpy *H*^ø^	− 39,3005		− 39,3022		− 39,3021	
20	*S*_vib_*·T*^ø^ contribution	9.6		9.7		9.5	
21	*S*_rot_*·T*^ø^ contribution	9.4		9.4		9.3	
22	*S*_trs_*·T*^ø^ contribution	12.4		12.4		12.4	
23	Total entropic term *S·T*^ø^	31.4		31.5		31.2	
24	Gibbs energy *G*^ø^ in opt str of L^0^	− 393,036		− 393,053		− 393,052	

^a^All energy quantities in units of kcal mol^−1^, 1 kcal mol^−1^ = 4.184 kJ mol^−1^; *debye*, *D* = 3.336 × 10^−30^ Ams; *angstrom*, *Å* = 10^−10^ m; *bohr*, *a*_0_ = 5.292 × 10^−11^ m; special units for polarizability α_*ij*_ = d*p*_*i*_/d*E*_*j*_: *α*(Å^3^) = 10^−24^ × *α*(cm^3^) = 0.1482 × 10^−24^ × *α*(*a*_0_^3^). Standard temperature *T*^ø^ = 298.15 K. Data for *p* and *Q* (isotropic value) in MP2 calculations refer to the relaxed electron density

The analysis of data listed in Table 1 for the zwitterionic form of the *l*-tyrosine brings these conclusions: (i) the zwiterionic (Z) form in water is more stable than the aminoacid (A1) form [items 4]; (ii) energies of HOMO, LUMO, vertical ionization energy, electron affinity, electronegativity and hardness are similar for the A- and Z-forms [items 1, 2, 6–9]; (iii) the dipole moment of the Z-form is more than 10-times higher than in the A-form [items 10]; (iv) the quadrupole moment and polarizability are similar for Z- and A-forms [items 11, 12]; (v) contributions from vibrations, rotations and translations to the inner energy and entropy are very similar [items 15–17, 20–23]; (vi) the *U*^ø^, *H*^ø^, *S*^ø^, and *G*^ø^ are almost identical.

The geometry optimization has been done also for the molecular cation (L^+^) and molecular anion (L^−^) of tyrosine (Table 2). Such a data allows evaluation of the adiabatic ionization energy, electron affinity, electronegativity, and hardness. Finally, after the vibrational analysis, the standard reaction Gibbs energy for the oxidation and/or reduction can be evaluated. Since the reaction Gibbs energy on reduction is negative, Δ_r_*G*^ø^(L^0^/L^−1^) =  − 10.6 kcal mol^−1^, *l*-tyrosine possesses a positive value of the absolute reduction potential.

**Table 2:** Calculated molecular properties of* l*-tyrosine in water by HF-MO-LCAO-SCF approximation^a^

Item	Molecule/ion	A1 form	Z form
3	*E*^+^ in optimized str of L^+^	− 393,050.41	− 393,049.79
4	*E*^0^ in optimized str of L^0^	− 393,159.42	− 393,160.09
5	*E*^−^ in optimized str of L^−^	− 393,163.41	− 393,165.38
6	Ionization energy *E*_i_ (a)	109	110
7	Electron affinity *E*_eg_ (a)	− 4.0	− 5.3
8	Molecular electronegativity χ_M_ (a)	56.5	57.8
9	Chemical hardness ƞ_P_ (a)	52.5	52.5
15	*E*_vib_(ZPE) – zero point energy	129.7	131.0
23	Total entropic term *S·T*^ø^	31.5	31.2
24	*G*^ø,+^ in optimized str of L^+^	− 39,2945.08	− 39,2942.86
25	*G*^ø,0^ in optimized str of L^0^	− 393,053.22	− 393,052.51
26	*G*^ø,*−*^ in optimized str of L^−^	− 393,062.70	− 393,063.14
27	Oxidation reaction Δ_r_^ø^*G*(L^0^/L^+^)	108.14	109.65
28	Reduction reaction Δ_r_^ø^*G*(L^0^/L^−^)	− 9.48	− 10.63
29	Oxidation potential *E*_abs_^ø^ (L^0^/L^+^) / V	− 4.69	− 4.75
30	Reduction potential *E*_abs_^ø^ (L^0^/L^−^) / V	+ 0.41	+ 0.46

^a^All energy quantities in units of kcal mol^−1^, 1 kcal mol^−1^ = 4.184 kJ mol^−1^; *debye*, *D* = 3.336 × 10^−30^ Ams; *angstrom*, *Å* = 10^−10^ m; *bohr*, *a*_0_ = 5.292 × 10^−11^ m; special units for polarizability α_*ij*_ = d*p*_*i*_/d*E*_*j*_: *α*(Å^3^) = 10^−24^ × *α*(cm^3^) = 0.1482 × 10^−24^ × *α*(*a*_0_^3^). Standard temperature *T*^ø^ = 298.15 K. Data for *p* and *Q* (isotropic value) in MP2 calculations refer to the relaxed electron density

The calculation procedure described above has been repeated using an alternate computational method, i.e., the density functional theory with the B3LYP hybrid functional of electron density. The results for the aminoacid form are presented in Table 3 and for the zwitterionic form in Table 5, both in water as a solvent (There are more detailed Tables S1 – S3 in Supplementary information.).*str* structure, *(v)* vertical ionization/affinity process. Ionization energy, *E*_i_ = *E*^+^  − *E*^0^; electron affinity, *E*_eg_ = *E*^−^  − *E*^0^; molecular electronegativity, χ_M_ = (*E*_i_ − *E*_eg_)/2; chemical hardness, ƞ_P_ = (*E*_i_ + *E*_eg_)/2; *(a)* adiabatic process

**Table 3:** Calculated molecular properties of* l*-tyrosine for the aminoacid and zwitterionic forms in water by DFT-B3LYP method using def2-TZVP basis set^a^

Item	Molecule/ion	Aminoacid A1 form	Aminoacid A2 form	Zwitterion Z form
3	*E*^+^ in optimized str of L^+^	− 395,158.01	− 395,158.23	− 395,157.89
4	*E*^0^ in optimized str of L^0^	− 395,289.78	− 395,292.76	− 395,293.28
5	*E*^−^ in optimized str of L^−^	− 395,317.05	− 395,315.44	− 395,318.16
6	Ionization energy *E*_i_ (a)	132	135	135
7	Electron affinity *E*_eg_ (a)	− 27.3	− 22.7	− 24.9
8	Molecular electronegativity χ_M_ (a)	79.6	78.8	80.1
9	Chemical hardness ƞ_P_ (a)	52.3	56.2	55.3
15	*E*_vib_(ZPE) – zero point energy	120.8	121.0	122.1
23	Total entropic term *S·T*^ø^	32.5	31.5	32.2
24	*G*^ø,+^ in optimized str of L^+^	− 395,061.30	− 395,061.25	− 395,059.95
25	*G*^ø,0^ in optimized str of L^0^	− 395,193.02	− 395,195.17	− 395,194.98
26	*G*^ø,−^ in optimized str of L^−^	− 395,223.30	− 395,222.81	− 395,224.25
27	Oxidation reaction Δ_r_*G*^ø^(L^0^/L^+^)	131.72	133.92	135.03
28	Reduction reaction Δ_r_*G*^ø^(L^0^/L^−^)	− 30.28	− 27.64	− 29.27
29	Oxidation potential *E*_abs_^ø^(L^0^/L^+^) / V	− 5.71	− 5.81	− 5.86
30	Reduction potential *E*_abs_^ø^(L^0^/L^*−*^) / V	+ 1.31	+ 1.20	+ 1.27

^a^All energy quantities in units of kcal mol^−1^, 1 kcal mol^−1^ = 4.184 kJ mol^−1^; *debye*, *D* = 3.336 × 10^−30^ Ams; *angstrom*, *Å* = 10^−10^ m; *bohr*, *a*_0_ = 5.292 × 10^−11^ m; special units for polarizability α_*ij*_ = d*p*_*i*_/d*E*_*j*_: *α*(Å^3^) = 10^−24^ × *α*(cm^3^) = 0.1482 × 10^−24^ × *α*(*a*_0_^3^). Standard temperature *T*^ø^ = 298.15 K. Data for *p* and *Q* (isotropic value) in MP2 calculations refer to the relaxed electron density

In both cases, the full geometry optimization was performed for the neutral molecule and its ions; this allows determining the energy difference for adiabatic processes. In the light of B3LYP calculations, the zwitterionic form (Z) in water is more stable relative to the aminoacid form A1 by Δ*E* = *E*^0^(Z) – *E*^0^(A1) =  − 3.5 kcal mol^−1^ and corresponding Δ*G*^ø^ = *G*^ø^(Z) – *G*^ø^(A1) =  − 1.96 kcal mol^−1^.

**Fig. 3:**
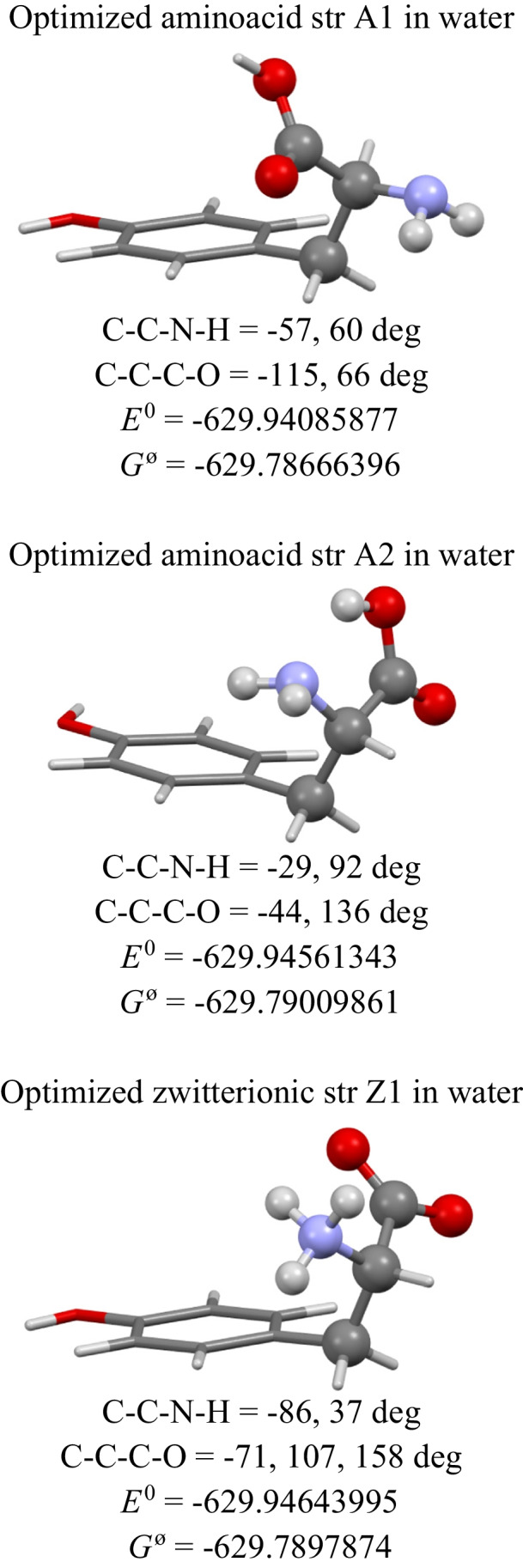
Optimized geometry for the aminoacid and zwitterionic forms of *l*-tyrosine by DFT-B3LYP method using def2-TZVP in water. Energetic data in *E*_h_

Recent report evaluated relative stability of four conformers of electroneutral *l*-tyrosine in vacuo, abbreviated as IICgg (at 0), IINgg (at 119 cm^−1^ = 0.34 kcal mol^−1^), IICg-g (at 180 cm^−1^ = 0.51 kcal mol^−1^) and IINg-g (at 185 cm^−1^ = 0.53 kcal mol^−1^); these are displayed in Figure S1 [27]. The coordinates of the most stable conformer IICgg with a five membered ring H^1^-O-C–C-NH_2_…H^1^ moiety have been used as an input for the full geometry optimization using the consistent method, basis set, and solvent as above resulting in the structure A2 (Fig. 3). Now, the energy differences are Δ*E* = *E*^0^(Z) – *E*^0^(A2) =  − 0.52 kcal mol^−1^ and Δ*G*^ø^ = *G*^ø^(Z) – *G*^ø^(A2) =  + 0.19 kcal mol^−1^.

**Fig. 4:**
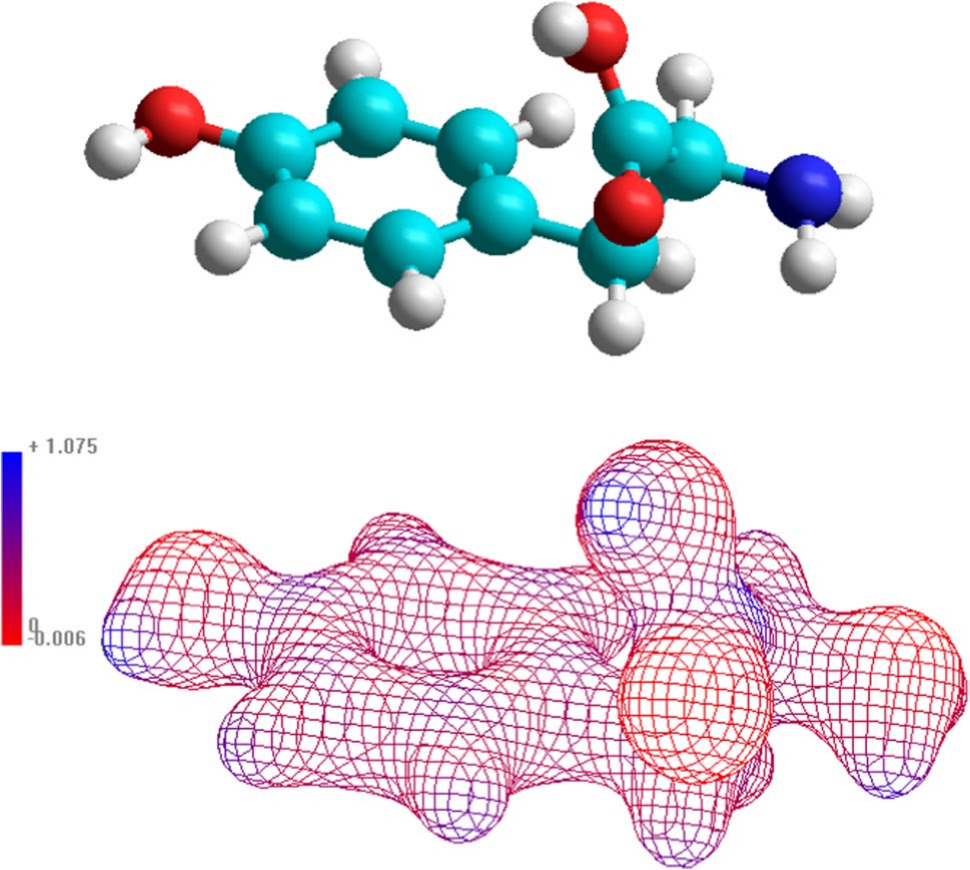
Optimized molecular structure for amino-form of *l*-tyrosine in vacuo, 3D mapped isosurface of charge density; contour 0.03 *ea*_0_^−1^, *a*_0_ – *bohr* unit. Blue – positive, red – negative

The molecular electrostatic potential is drawn in Fig. 4 along with the optimized molecular structure (HF-SCF level) in vacuo. This 3D figure can be helpful in understanding the docking of *l*-tyrosine [28, 29].

Simple QSAR calculations (quantitative structure–activity relationships) based upon additive increments gave the following molecular descriptors of *l*-tyrosine [18]: volume *V* = 559 Å^3^, surface area *S* = 362 Å^2^, hydration energy − 18.09 kcal mol^−1^, molar refractivity *R* = 22.4 Å^3^, and octanol–water partition coefficient log*P* = 2.77 (The polarizability estimated from an additivity scheme cannot distinguish isomers of any type [30].).

The Pearson hardness reflects the resistance of the molecule against the electron transfer and for the *l*-tyrosine it is 108 and 123 kcal mol^−1^ in vacuo by ΔSCF and MP2 calculations, respectively. Upon solvation in water, these data are reduced to 59 and 76 kcal mol^−1^ (data for the aminoacid form). Table 4 brings a comparison of the molecular descriptors calculated by various methods for aminoacid as well as zwitterionic forms of *l*-tyrosine. It is registered that the calculated absolute reduction potential *E*_abs_^ø^(L^0^/L^−^) (when available from the adiabatic affinity processes) correlates with the electrophilicity index ω. These quantities for the ΔSCF(a) calculations are heavily underestimated (about 0.43 V) relative to the B3LYP calculations (about 1.25 V).

**Table 4:** Review of molecular descriptors for *l*-tyrosine in water^a^

def2-TZVP	Ionization energy *E*_ion_	Electron affinity *E*_eg_	Electronegativity χ	Hardness ƞ	Electrophylicity ω	Dipole moment *p*	Polarizability α	Reduction potential *E*_red_^ø^
*Aminoacid form A1*								
ΔSCF (v)	117	2	57	59	28	2.189	151.6	
ΔSCF + MP2 (v)	160	-8	84	76	46	2.148	160.9	
ΔSCF (a)	109	− 4.0	56	52	30	2.189	151.6	0.41
B3LYP (a)	132	− 27.3	80	52	61	1.798	169.6	1.31
*Aminoacid form A2*								
B3LYP (a)	135	− 22.7	79	56	55	5.415	168.3	1.20
*Zwitterionic form*								
ΔSCF (v)	118	1.3	59	60	29	16.31	150.9	
ΔSCF + MP2 (v)	163	− 8.3	85	77	47	15.72	160.8	
ΔSCF (a)	110	− 5.3	58	52	32	16.31	150.9	0.46
B3LYP (a)	135	− 24.9	80	55	58	15.60	168.7	1.27

^a^Units as in Table 1

* (v)* vertical process, *(a)* adiabatic process

Electronegativity, hardness, dipole moment, and polarizability have been selected for the comparison along the series of aminoacids, monoaminergic neurotransmitters, trace amines, and related drugs (Table 5). These descriptors reflect unique collective properties of molecules. Of several methods, ΔSCF is a rather weak approximation that ignores the correlation energy. The most reliable are B3LYP(a) results that account to the correlation energy and the electron and nuclear relaxation during the ionization/affinity processes.

**Table 5:**
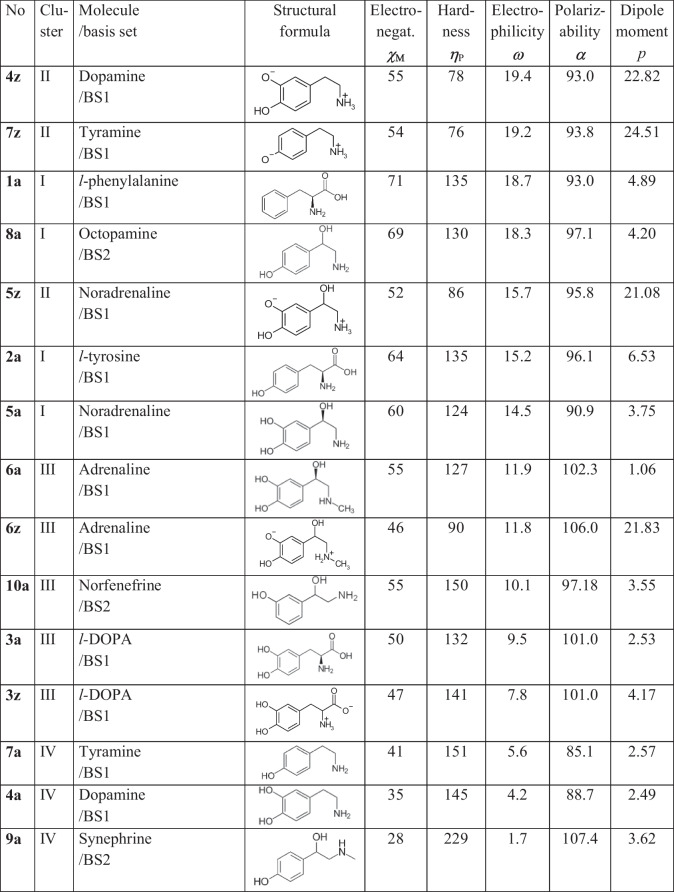
Molecular descriptors calculated at MP2 level for related phenethylamines, catecholamines, and α-aminoacids^a^

^a^Preliminary data by MP2 using BS1 = 631G^**^, BS2 = TZVP basis sets at optimized geometry in vacuo.

For units, see footnote to Table 1. Data sorted according to the electrophilicity index

**Fig. 5:**
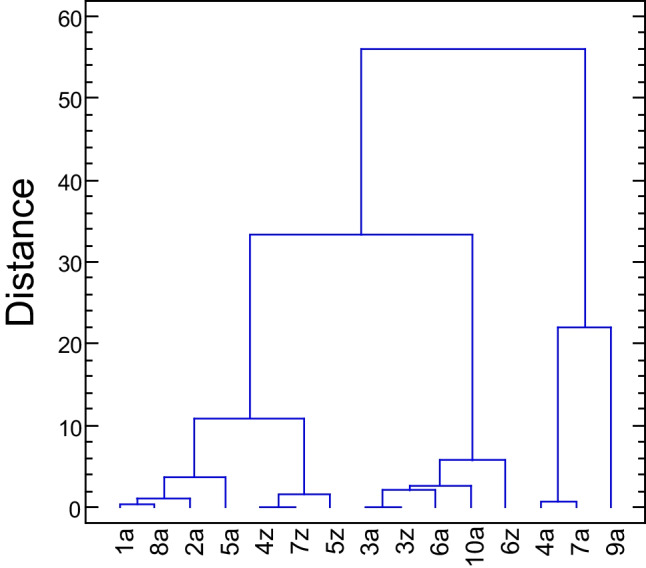
Cluster analysis (Wards method) showing similarity of species in Table 5 based upon molecular electronegativity, chemical hardness, electrophilicity and polarizability calculated at MP2 level. Numbering of species according to Table 5

A similarity of the species listed in Table 5 can be evaluated by the cluster analysis (Fig. 5): Wards method and Euclidean norm show that according to the “distance” the whole set is split into three clusters [31]. The selected descriptors of the target molecule No **2a** are similar to species **1a**, **8a**, and eventually **5a** (aminoacid forms of phenylalanine, octopamine and noradrenaline, cluster I); a significant degree of similarity represents the set of **4z**, **5z**, and **7z** (zwitterionic forms of dopamine, noradrenaline and tyramine, cluster II); there is some similarity with the cluster formed of **3a**, **3z**, **6a**, and **10a** (*l*-DOPA, adrenaline and norfenefrine, cluster III); they are very different from **4a**, **7a**, and **9a** (aminocid forms of dopamine, tyramine and synephrine, cluster IV). The classification into clusters correlates with the value of the electrophilicity index: ω > 14 refers to clusters I and II, ω < 6 is characteristic for the cluster IV.

## Conclusions

On conclusions*, *ab initio HF-MO-LCAO-SCF + MP2 calculations show that the molecule of *l*-tyrosine possesses stationary structural forms — aminoacid (A1) and zwitterionic (Z). In the polar solvent like water they differ only slightly in the total energy: the Z-form is more stable by *E*^0^(Z) – *E*^0^(A1) = 1 and 4 kcal mol^−1^ for SCF level and MP2 correction, respectively. Their standard Gibbs energy is almost identical, *G*^ø^(A1) ~ *G*^ø^(Z) at SCF level. The solvation reduces the electron affinity and alters its sign from positive to negative values; this lowers the chemical hardness as well. The dipole moment dramatically differs for the aminoacid and Z-forms due to a large separation of charged sites. Of applied methods, B3LYP(a) results are the most reliable because they account to the correlation energy and the electron and nuclear relaxation during the ionization/affinity processes.

The second stationary geometry A2 contains a five membered ring H^1^-O-C–C-N(H_2_)…H^1^ and it is more stable relative to the conformer A1 (suggested by PubChem CID 6057) by Δ*G*^ø^ = *G*^ø^(A1) – *G*^ø^(A2) =  − 2.1 kcal mol^−1^. The standard Gibbs energy is almost the same as for the zwitterionic form (suggested by CCDC 1208550): Δ*G*^ø^ = *G*^ø^(Z) – *G*^ø^(A2) =  + 0.2 kcal mol^−1^. According to B3LYP calculations, the absolute reduction potential is *E*_red_^ø^ = 1.31, 1.20, and 1.27 V for A1, A2, and Z forms, respectively. The cluster analysis confirms that *l*-tyrosine (aminoacid) is mostly similar to *l*-phenylalanine (aminoacid differing in one –OH group), octopamine (amine), and noradrenaline (amine). The norfenefrine (amine), an isomer of octopamine, spans another cluster of similar compounds: *l*-DOPA (aminoacid) and adrenaline (amine).

## Supplementary Information

Below is the link to the electronic supplementary material.

Supplementary file1 (DOCX 250 KB)

## Data Availability

The datasets generated during and/or analyzed during the current study are available from the corresponding author on reasonable request.
