# Supplementary material for: Ab initio study of molecular properties of l-tyrosine
Source: J Mol Model. 2023 Jul 13;29(8):245. doi: 10.1007/s00894-023-05648-8 (PMC10344843; doi:10.1007/s00894-023-05648-8)
Supplement: Supplementary file 1 — Supplementary file1 (DOCX 250 KB) [file 894_2023_5648_MOESM1_ESM.docx]

Ab initio study of molecular properties of *l*-tyrosine

Roman Boča,^*^ Juraj Štofko, Richard Imrich

Faculty of Health Sciences, University of SS Cyril and Methodius, 91701 Trnava, Slovakia

***Supplementary information***

**Table S1**. Energetic quantities at MO-LCAO-SCF approximation calculated for the optimized structures of the zwitterionic forms of *l*-tyrosine in water (enlarged dataset). ^a^

| def2-TZVP basis set | | In optimized geometry | | |
| --- | --- | --- | --- | --- |
| Item, *molecular properties* | | L^+^ | L^0^ (A1) | L^-^ |
| 1 | Energy of HOMO |  | -197 |  |
| 2 | Energy of LUMO |  | 73 |  |
| 3-5 | Energy *E^q^* in optimized geometry | -393049.79 | -393160.09 | -393165.38 |
| 6 | Ionization energy, *E*_i_ (a) |  | 110.30 |  |
| 7 | Electron affinity, *E*_eg_ (a) |  | -5.29 |  |
| 8 | Molecular electronegativity, *χ*_M_ (a) |  | 57.8 |  |
| 9 | Chemical hardness, *η*_P_ (a) |  | 52.5 |  |
| 10 | Dipole moment *p* / debye | undefined | 16.308 | undefined |
| 11 | Quadrupole moment *Q* / e*a*_0_^2^ | -41.25 | -55.78 | -69.23 |
| 12 | Dipole polarizability *α */ *a*_0_^3^ |  | 150.92 |  |
| 13 | Solvated surface area *S* / *a*_0_^2^ | 789 | 784 | 789 |
| 14 | Solvated volume *V* / *a*_0_^3^ | 1451 | 1441 | 1442 |
| *Vibrational analysis and thermodynamic functions at standard conditions* | | | | |
| 15 | *E*_vib_(ZPE) – zero point energy | 130.78 | 130.98 | 126.37 |
| 16 | Overall *E*_vib_(*T*^ø^) contribution | 136.27 | 136.46 | 132.38 |
| 17 | *E*_rot_ = *E*_trs_ contribution | 0.89 | 0.89 | 0.89 |
| 18 | Inner energy *U*^ø^ | -392911.74 | -393021.86 | -393031.22 |
| 19 | Enthalpy *H*^ø^ | -392911.15 | -393021.26 | -393030.63 |
| 20 | *S*_vib_*·T*^ø^ contribution | 9.59 | 9.53 | 10.37 |
| 21 | S_rot_*·T*^ø^ contribution | 9.34 | 9.34 | 9.36 |
| 22 | S_trs_*·T*^ø^ contribution | 12.37 | 12.37 | 12.37 |
| 23 | Total entropic term *S·T*^ø^ | 31.71 | 31.25 | 32.51 |
| 24 | Gibbs energy *G*^ø^ | -392942.86 | -393052.51 | -393063.14 |
| 25 | *G*^ø^ – *E*^0^ | 106.94 | 107.58 | 102.25 |
| 26 | Δ_r_*G*^ø^ = *G^q^* – *G*^0^ | ox: 109.65 | ← → | red: -10.63 |
| 27 | Redox potential *E*_abs_^ø^(L^0^/L*^q^*) / V | ox: -4.75 | ← → | red: +0.46 |

**Table S2.** Calculated molecular properties of *l*-tyrosine for the **aminoacid** form in water by DFT-B3LYP method (enlarged data set). ^a^

| def2-TZVP basis set | | In optimized geometry | | |
| --- | --- | --- | --- | --- |
| Item, *molecular properties* | | L^+^ | L^0^ (A1) | L^-^ |
| 1 | Energy of HOMO |  | 140 |  |
| 2 | Energy of LUMO |  | -11.1 |  |
| 3-5 | Energy *E^q^* in optimized geometry | -395158.01 | -395289.78 | -395317.05 |
| 6 | Ionization energy, *E*_i_ (a) |  | 132 |  |
| 7 | Electron affinity, *E*_eg_ (a) |  | -27.3 |  |
| 8 | Molecular electronegativity, *χ*_M_ (a) |  | 79.6 |  |
| 9 | Chemical hardness, *η*_P_ (a) |  | 52.3 |  |
| 10 | Dipole moment *p* / debye | undefined | 1.798 | undefined |
| 11 | Quadrupole moment *Q* / e*a*_0_^2^ |  | -56.11 |  |
| 12 | Dipole polarizability *α */ *a*_0_^3^ |  | 169.6 |  |
| 13 | Solvated surface area *S* / *a*_0_^2^ | 796 | 812 | 818 |
| 14 | Solvated volume *V* / *a*_0_^3^ | 1453 | 1464 | 1483 |
| *Vibrational analysis and thermodynamic functions at standard conditions* | | | | |
| 15 | *E*_vib_(ZPE) – zero point energy | 120.79 | 120.83 | 118.30 |
| 16 | Overall *E*_vib_(*T*^ø^) contribution | 126.81 | 126.88 | 124.66 |
| 17 | *E*_rot_ = *E*_trs_ contribution | 0.89 | 0.89 | 0.89 |
| 18 | Inner energy *U*^ø^ | -395029.42 | -395161.13 | -395190.62 |
| 19 | Enthalpy *H*^ø^ | -395028.83 | -395160.54 | -395190.02 |
| 20 | *S*_vib_*·T*^ø^ contribution | 10.35 | 10.74 | 11.04 |
| 21 | S_rot_*·T*^ø^ contribution | 9.35 | 9.38 | 9.42 |
| 22 | S_trs_*·T*^ø^ contribution | 12.37 | 12.37 | 12.37 |
| 23 | Total entropic term *S·T*^ø^ | 32.47 | 32.49 | 33.28 |
| 24 | Gibbs energy *G*^ø^ | -395061.30 | -395193.02 | -395223.30 |
| 25 | *G*^ø^ – *E*^0^ | 96.71 | 96.76 | 93.75 |
| 26 | Δ_r_*G*^ø^ = *G^q^* – *G*^0^ | ox: 131.72 | ← → | red: -30.28 |
| 27 | Redox potential *E*_abs_^ø^(L^0^/L*^q^*) / V | ox: -5.71 | ← → | ox: +1.31 |

**Table S3.** Calculated molecular properties of *l*-tyrosine for the **zwitterionic** form in water by DFT-B3LYP method (enlarged data set). ^a^

| def2-TZVP basis set | | In optimized geometry | | |
| --- | --- | --- | --- | --- |
| Item, *molecular properties* | | L^+^ | L^0^ (Z) | L^-^ |
| 1 | Energy of HOMO |  | 143 |  |
| 2 | Energy of LUMO |  | -11.3 |  |
| 3-5 | Energy *E^q^* in optimized geometry | -395157.89 | -395293.28 | -395318.16 |
| 6 | Ionization energy, *E*_i_ (a) |  | 135.39 |  |
| 7 | Electron affinity, *E*_eg_ (a) |  | -24.87 |  |
| 8 | Molecular electronegativity, *χ*_M_ (a) |  | 80.1 |  |
| 9 | Chemical hardness, *η*_P_ (a) |  | 55.3 |  |
| 10 | Dipole moment *p* / debye | undefined | 15.600 | undefined |
| 11 | Quadrupole moment *Q* / e*a*_0_^2^ |  | -55.40 |  |
| 12 | Dipole polarizability *α */ *a*_0_^3^ |  | 168.7 |  |
| 13 | Solvated surface area *S* / *a*_0_^2^ | 790 | 794 | 782 |
| 14 | Solvated volume *V* / *a*_0_^3^ | 1458 | 1453 | 1449 |
| *Vibrational analysis and thermodynamic functions at standard conditions* | | | | |
| 15 | *E*_vib_(ZPE) – zero point energy | 122.17 | 122.12 | 117.82 |
| 16 | Overall *E*_vib_(*T*^ø^) contribution | 128.10 | 128.03 | 123.85 |
| 17 | *E*_rot_ = *E*_trs_ contribution | 0.89 | 0.89 | 0.89 |
| 18 | Inner energy *U*^ø^ | -395028.01 | -395163.48 | -395192.53 |
| 19 | Enthalpy *H*^ø^ | -395027.42 | -395162.88 | -395191.93 |
| 20 | *S*_vib_*·T*^ø^ contribution | 10.42 | 10.38 | 10.18 |
| 21 | S_rot_*·T*^ø^ contribution | 9.33 | 9.34 | 9.36 |
| 22 | S_trs_*·T*^ø^ contribution | 12.37 | 12.37 | 12.37 |
| 23 | Total entropic term *S·T*^ø^ | 32.53 | 32.19 | 32.32 |
| 24 | Gibbs energy *G*^ø^ | -395059.95 | -395194.98 | -395224.25 |
| 25 | *G*^ø^ – *E*^0^ | 97.94 | 98.30 | 93.91 |
| 26 | Δ_r_*G*^ø^ = *G^q^* – *G*^0^ | ox: 135.03 | ← → | red: -29.27 |
| 27 | Redox potential *E*_abs_^ø^(L^0^/L*^q^*) / V | ox: -5.86 | ← → | red: +1.27 |

| 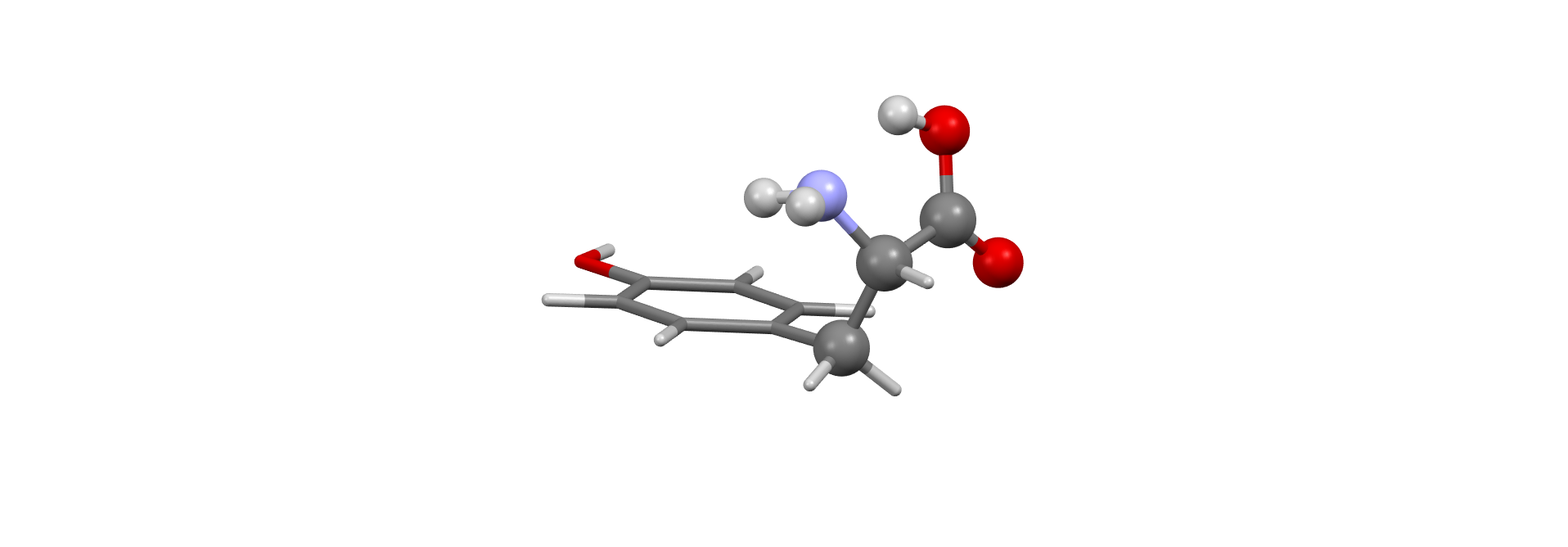  IICgg, *E*^0^ = -629.**1488**8686 | 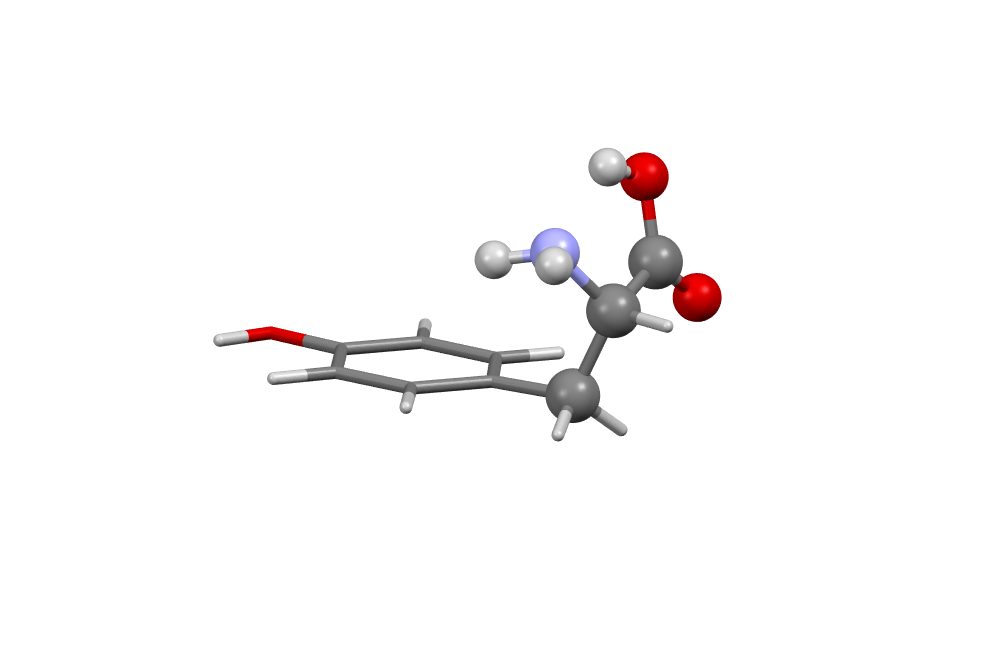  IINgg, *E*^0^ = -629.**1483**5160 |
| --- | --- |
| 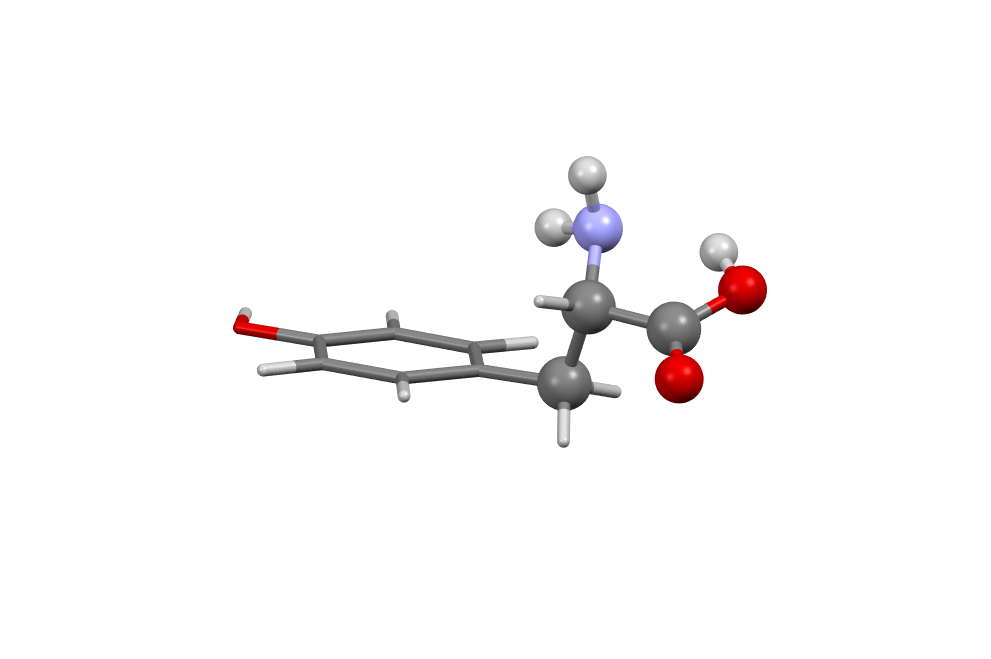  IICg-g, *E*^0^ = -629.**14762**038 | 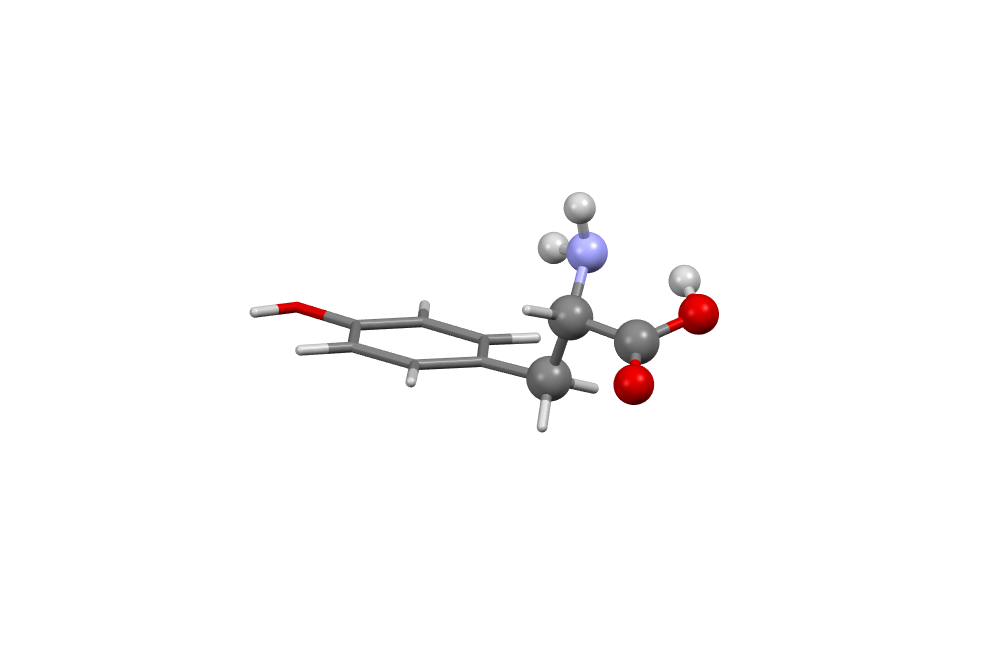  IINg-g, *E*^0^ = -629.**14763**052 |

**Figure S1** Literature data for the conformers of the aminoacid form of *l*-tyrosine *in vacuo* [27]. Critical digits are bold-typed. All data in E_h_.

Table S4. Calculated vibrational frequencies for the aminoacid form A1 in water by B3LYP

Mode freq eps Int T**2 TX TY TZ

cm**-1 L/(mol*cm) km/mol a.u.

----------------------------------------------------------------------------

6: **11.16** 0.001222 6.17 0.034167 (-0.154189 0.091356 -0.045237)

7: 34.92 0.000553 2.79 0.004938 (-0.038526 -0.023911 -0.053685)

8: 50.02 0.000386 1.95 0.002407 (-0.039269 0.010091 -0.027622)

9: 77.55 0.000054 0.27 0.000216 (-0.014384 -0.002333 -0.001989)

10: 147.65 0.000016 0.08 0.000033 (-0.002217 0.004878 -0.002111)

11: 233.15 0.010314 52.12 0.013805 (-0.013737 -0.105683 0.049475)

12: 241.30 0.003262 16.48 0.004219 ( 0.034089 0.042428 -0.035445)

13: 283.84 0.002073 10.48 0.002279 (-0.023759 0.035566 -0.021205)

14: 301.68 0.004373 22.10 0.004524 ( 0.016579 0.042581 -0.049353)

15: 329.35 0.000844 4.26 0.000800 ( 0.016260 -0.021827 -0.007666)

16: 363.81 0.034110 172.38 0.029258 ( 0.051791 0.098643 0.129790)

17: 373.95 0.001410 7.13 0.001177 (-0.010258 -0.009612 -0.031290)

18: 426.48 0.000049 0.25 0.000036 ( 0.004825 -0.003445 -0.000648)

19: 434.47 0.002530 12.78 0.001817 ( 0.040760 -0.010062 0.007382)

20: 472.38 0.004259 21.52 0.002813 (-0.031044 0.025464 0.034660)

21: 526.37 0.009209 46.54 0.005460 ( 0.017728 0.045754 0.055244)

22: 557.28 0.004305 21.76 0.002411 (-0.015813 0.028170 0.036976)

23: 620.92 0.022306 112.73 0.011211 ( 0.093788 -0.026640 0.041290)

24: 637.29 0.010820 54.68 0.005298 ( 0.042596 0.048287 0.033941)

25: 656.08 0.000334 1.69 0.000159 ( 0.005497 -0.009728 -0.005834)

26: 733.80 0.000887 4.48 0.000377 (-0.014925 0.009877 0.007548)

27: 769.11 0.025412 128.42 0.010311 ( 0.092525 -0.035593 0.021981)

28: 789.93 0.004954 25.04 0.001957 (-0.042488 -0.008951 -0.008480)

29: 800.42 0.001827 9.24 0.000712 ( 0.024330 -0.009053 0.006211)

30: 831.17 0.001303 6.59 0.000489 ( 0.008326 0.013053 0.015796)

31: 848.15 0.014772 74.65 0.005435 (-0.022865 -0.040123 -0.057466)

32: 862.96 0.008625 43.59 0.003119 ( 0.040411 0.016637 0.034775)

33: 899.46 0.040749 205.93 0.014138 ( 0.114581 -0.007630 0.030832)

34: 954.48 0.001863 9.41 0.000609 ( 0.024530 0.002665 -0.000341)

35: 966.59 0.001051 5.31 0.000339 ( 0.010854 0.006625 -0.013331)

36: 972.06 0.000145 0.73 0.000047 (-0.005157 0.000542 -0.004435)

37: 1031.40 0.000140 0.71 0.000042 (-0.003518 -0.003321 -0.004340)

38: 1038.18 0.014462 73.08 0.004347 ( 0.057387 0.001142 0.032442)

39: 1102.85 0.013899 70.24 0.003933 ( 0.005688 0.035474 0.051402)

40: 1120.56 0.008217 41.52 0.002288 ( 0.018737 -0.026027 0.035493)

41: 1138.62 0.068732 347.34 0.018837 ( 0.029844 -0.069155 -0.114736)

42: 1168.40 0.009780 49.42 0.002612 ( 0.048465 0.000962 -0.016193)

43: 1186.68 0.060029 303.36 0.015786 (-0.114819 0.039069 0.032803)

44: 1193.76 0.000331 1.67 0.000086 (-0.005326 -0.002038 0.007341)

45: 1225.22 0.001642 8.30 0.000418 ( 0.015829 0.009353 0.008951)

46: 1227.58 0.005573 28.17 0.001417 (-0.003056 -0.024482 -0.028427)

47: 1272.01 0.030675 155.02 0.007526 (-0.079745 0.031923 0.012130)

48: 1309.54 0.003563 18.01 0.000849 (-0.006270 -0.021519 -0.018621)

49: 1330.21 0.001099 5.55 0.000258 ( 0.004187 0.005567 -0.014468)

50: 1346.99 0.004499 22.74 0.001042 (-0.011000 0.029022 0.008887)

51: 1363.02 0.001639 8.28 0.000375 ( 0.018780 0.004049 0.002481)

52: 1367.80 0.005963 30.13 0.001360 ( 0.030781 -0.018114 -0.009209)

53: 1442.17 0.006514 32.92 0.001410 (-0.007812 0.022102 0.029326)

54: 1467.07 0.004541 22.95 0.000966 (-0.027542 0.011717 0.008378)

55: 1474.99 0.003616 18.27 0.000765 ( 0.013365 0.008369 0.022723)

56: 1541.28 0.035795 180.89 0.007247 ( 0.078913 -0.027925 -0.015503)

57: 1624.41 0.015508 78.37 0.002979 (-0.018984 0.022457 0.045984)

58: 1626.01 0.007650 38.66 0.001468 (-0.029720 -0.002753 0.024029)

59: 1646.11 0.013095 66.17 0.002482 (-0.044593 0.021249 0.006513)

60: 1757.24 0.102785 519.44 0.018253 (-0.060953 -0.053933 0.107839)

61: 3033.89 0.007549 38.15 0.000776 (-0.014807 0.017519 0.015820)

62: 3071.66 0.001249 6.31 0.000127 (-0.001657 0.011026 -0.001596)

63: 3089.26 0.006486 32.78 0.000655 ( 0.009716 0.009218 -0.021813)

64: 3156.67 0.003085 15.59 0.000305 ( 0.015844 -0.007058 -0.002019)

65: 3159.17 0.002141 10.82 0.000212 (-0.007739 0.011484 -0.004445)

66: 3173.61 0.002643 13.36 0.000260 ( 0.005650 0.010661 -0.010692)

67: 3179.76 0.001443 7.29 0.000142 (-0.007151 -0.006450 0.006994)

68: 3470.13 0.000796 4.02 0.000072 ( 0.005611 -0.003679 -0.005155)

69: 3541.40 0.002964 14.98 0.000261 (-0.001956 -0.014077 0.007695)

70: 3674.65 0.026192 132.36 0.002224 (-0.004549 -0.045193 -0.012699)

71: 3760.41 0.036456 184.24 0.003025 ( 0.034556 -0.040363 0.014215)

* The epsilon (eps) is given for a Dirac delta lineshape.

** The dipole moment derivative (T) already includes vibrational overlap.

The first frequency considered to be a vibration is 6 (modes No 0-5 refer to molecular rotation and translation). The total number of vibrations considered is 66

Table S5. Calculated vibrational frequencies for the zwitterionic form in water by B3LYP

Mode freq eps Int T**2 TX TY TZ

cm**-1 L/(mol*cm) km/mol a.u.

----------------------------------------------------------------------------

6: **23.71** 0.004453 22.50 0.058598 ( 0.016450 0.197118 -0.139543)

7: 40.38 0.004569 23.09 0.035312 (-0.046417 0.180490 -0.024095)

8: 64.86 0.001035 5.23 0.004978 (-0.045641 -0.047408 0.025445)

9: 85.01 0.000402 2.03 0.001476 (-0.013901 -0.014873 -0.032588)

10: 125.72 0.002283 11.54 0.005666 (-0.002325 0.056547 0.049629)

11: 174.67 0.001517 7.67 0.002710 ( 0.028973 -0.007335 -0.042625)

12: 217.59 0.006950 35.12 0.009967 (-0.060497 -0.072126 -0.033245)

13: 302.14 0.004567 23.08 0.004718 (-0.010110 -0.005895 0.067680)

14: 321.24 0.001660 8.39 0.001612 ( 0.012975 0.025157 0.028480)

15: 360.67 0.005033 25.44 0.004355 (-0.043277 -0.006857 0.049347)

16: 368.02 0.034743 175.58 0.029460 (-0.164326 -0.026046 0.042180)

17: 418.52 0.001351 6.82 0.001007 (-0.006952 -0.008516 -0.029768)

18: 426.78 0.000804 4.06 0.000588 ( 0.005339 -0.023554 0.002173)

19: 434.28 0.002604 13.16 0.001871 ( 0.010412 0.041089 -0.008641)

20: 488.21 0.003528 17.83 0.002255 ( 0.005480 -0.022216 0.041612)

21: 517.31 0.007426 37.53 0.004480 ( 0.054104 0.012882 0.037237)

22: 579.45 0.007791 39.37 0.004196 (-0.053147 0.004526 0.036751)

23: 630.52 0.000586 2.96 0.000290 (-0.014941 -0.001113 0.008094)

24: 655.58 0.000178 0.90 0.000085 (-0.003079 -0.008307 -0.002491)

25: 724.38 0.002976 15.04 0.001282 ( 0.000235 0.033673 0.012177)

26: 750.08 0.004065 20.54 0.001691 (-0.008460 -0.039701 -0.006585)

27: 795.22 0.002849 14.40 0.001118 (-0.018062 0.013194 -0.024853)

28: 831.84 0.001300 6.57 0.000488 (-0.020326 -0.001323 0.008529)

29: 839.60 0.007524 38.02 0.002797 (-0.022186 -0.020152 0.043570)

30: 848.92 0.018824 95.13 0.006920 (-0.062338 0.011188 0.053930)

31: 865.44 0.006885 34.79 0.002483 ( 0.026599 0.023083 0.035247)

32: 890.00 0.007767 39.25 0.002724 ( 0.034538 0.038998 -0.003132)

33: 942.51 0.004295 21.71 0.001422 ( 0.012595 0.017038 -0.031197)

34: 965.83 0.000602 3.04 0.000195 (-0.002543 -0.000176 -0.013717)

35: 977.92 0.000044 0.22 0.000014 ( 0.001177 0.000889 0.003457)

36: 1015.75 0.004652 23.51 0.001429 (-0.017717 -0.007728 -0.032491)

37: 1032.05 0.000127 0.64 0.000039 ( 0.000854 -0.005912 -0.001685)

38: 1094.24 0.005227 26.42 0.001491 ( 0.027018 -0.021016 -0.017861)

39: 1115.57 0.006831 34.52 0.001911 (-0.043091 -0.006749 0.002911)

40: 1130.63 0.009876 49.91 0.002726 ( 0.000084 -0.038450 0.035320)

41: 1179.12 0.060644 306.47 0.016050 ( 0.009698 -0.122872 -0.029298)

42: 1197.43 0.001023 5.17 0.000267 (-0.000188 -0.014408 0.007673)

43: 1213.94 0.004002 20.23 0.001029 (-0.005933 0.028663 0.013119)

44: 1229.22 0.003384 17.10 0.000859 ( 0.017399 -0.019441 -0.013359)

45: 1274.15 0.028371 143.38 0.006949 (-0.009834 0.082518 0.006536)

46: 1292.23 0.002669 13.49 0.000645 ( 0.007163 0.021031 -0.012287)

47: 1340.52 0.008686 43.90 0.002022 ( 0.011952 -0.016891 -0.039925)

48: 1348.87 0.024099 121.79 0.005575 ( 0.018753 0.012971 -0.071102)

49: 1366.53 0.009432 47.66 0.002154 (-0.010917 0.037388 0.025234)

50: 1392.31 0.004968 25.11 0.001113 ( 0.015475 0.028892 0.006264)

51: 1399.96 0.023735 119.95 0.005291 ( 0.057705 -0.012573 -0.042460)

52: 1463.27 0.067567 341.46 0.014410 (-0.081023 0.042869 -0.077506)

53: 1467.93 0.003802 19.21 0.000808 (-0.002405 -0.028048 -0.003975)

54: 1475.69 0.012492 63.13 0.002642 ( 0.011707 -0.014910 0.047774)

55: 1542.46 0.034959 176.67 0.007073 ( 0.008499 -0.082729 -0.012500)

56: 1601.89 0.028264 142.83 0.005506 ( 0.004122 -0.070536 0.022668)

57: 1624.59 0.006168 31.17 0.001185 (-0.001334 0.023740 0.024889)

58: 1643.67 0.160832 812.78 0.030535 (-0.146174 0.078126 -0.055361)

59: 1646.36 0.007830 39.57 0.001484 (-0.012100 -0.036568 -0.000742)

60: 1654.46 0.017920 90.56 0.003380 (-0.015172 0.027610 0.048862)

61: 3041.81 0.005824 29.43 0.000598 ( 0.012166 -0.013097 -0.016672)

62: 3092.09 0.001891 9.56 0.000191 ( 0.006793 -0.005320 0.010790)

63: 3109.93 0.003134 15.84 0.000314 ( 0.004917 -0.017038 0.000041)

64: 3156.67 0.002753 13.91 0.000272 ( 0.000015 -0.014303 -0.008219)

65: 3169.25 0.002256 11.40 0.000222 ( 0.004638 -0.012447 0.006761)

66: 3170.90 0.001258 6.36 0.000124 (-0.002625 0.002447 -0.010534)

67: 3185.00 0.001501 7.59 0.000147 (-0.003476 -0.003409 -0.011107)

68: 3365.32 0.017134 86.59 0.001589 ( 0.026700 -0.022360 -0.019389)

69: 3463.27 0.028189 142.46 0.002540 ( 0.001628 0.026740 0.042689)

70: 3500.64 0.027209 137.50 0.002426 ( 0.031388 0.034122 -0.016615)

71: 3760.32 0.034938 176.56 0.002899 ( 0.013706 -0.045029 0.026154)

Units conversions

|  | hartree | eV | cm^-1^ | kcal/mol | kJ/mol |
| --- | --- | --- | --- | --- | --- |
| hartree | 1 | 27.2107 | 219 474.63 | 627.503 | 2 625.5 |
| eV | 0.0367502 | 1 | 8 065.73 | 23.060 9 | 96.486 9 |
| cm^-1^ | 4.556 33 x 10^-6^ | 1.239 81 x 10^-4^ | 1 | 0.002 859 11 | 0.011 962 7 |
| kcal/mol | 0.001 593 62 | 0.043 363 4 | 349.757 | 1 | 4.18400 |
| kJ/mol | 0.000 380 88 | 0.010 364 10 | 83.593 | 0.239001 | 1 |
